# The Connection between Space and Thinking: An Interview with Rafael Viñoly

**DOI:** 10.1371/journal.pgen.1002445

**Published:** 2011-12-29

**Authors:** Jane Gitschier

**Affiliations:** Department of Medicine and Pediatrics, University of California San Francisco, San Francisco, California, United States of America

**Image 1:**
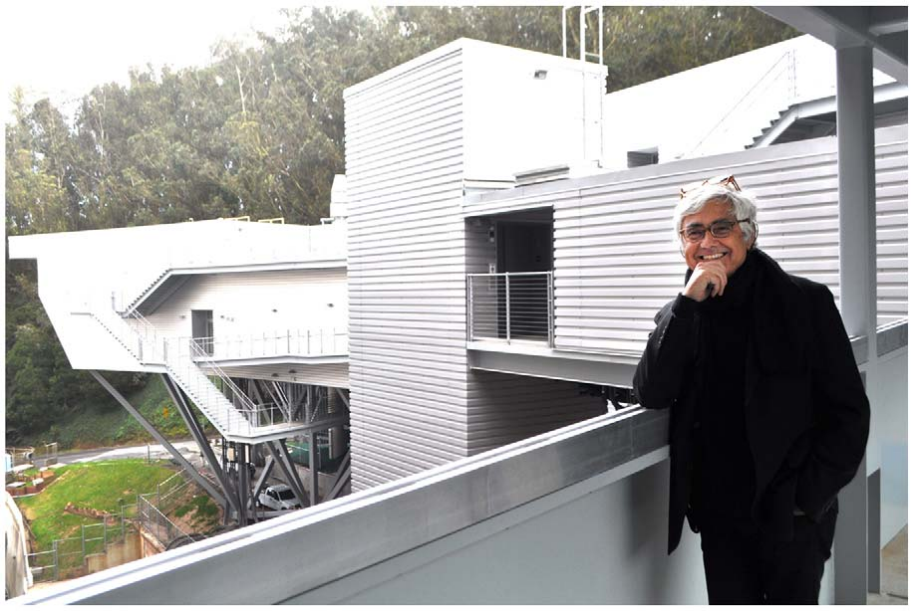
Rafael Viñoly at the new Ray and Dagmar Dolby Regeneration Medicine Building. Photograph courtesy of Michael Toporkoff, University of California San Francisco.

Three years ago, a member of my university's project management team came by my office with a set of Bose noise-canceling headphones and some advice: “You're going to need these.” And indeed I did, as just outside my ninth floor window, on the steep slope of Mt. Sutro at the back-side of the University of California San Francisco's (UCSF) Parnassus Campus, a laboratory for stem cell research was about to be constructed. For two-and-half years, I wore those headphones and witnessed, at eye-level, the construction of an unusually elegant and stunningly situated laboratory, all the while trying to secure an interview with its Uruguayan-American architect, Rafael Viñoly (Image 1).

**Image 2:**
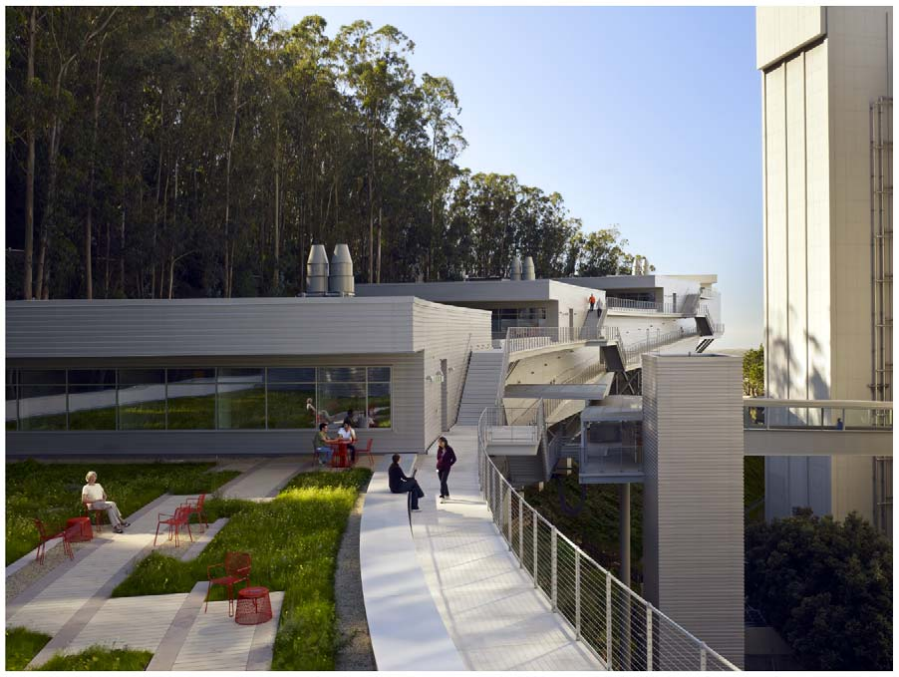
The Ray and Dagmar Dolby Regeneration Medicine Building at the University of California San Francisco. Photograph courtesy of Bruce Damonte and Rafael Viñoly Architects. More pictures of the building can be seen at the Rafael Viñoly Architects website: http://www.rvapc.com/.

I had first learned of Viñoly when I visited Princeton to interview David Botstein for *PLoS Genetics*. Viñoly had designed the Carl Icahn Laboratory there, and I was struck by its expansive atrium with its cleverly filtered light. Viñoly had also designed the crown jewel of all laboratories, Howard Hughes Medical Institute's (HHMI) Janelia Farm in Virginia. When I saw the renderings for the UCSF building, I was flabbergasted. The plan called for a building of five stories, each gently fanning out from the one below it and supporting a rooftop garden for the next one in the series. The building hugs the upward curve of Medical Center Way and gives the feeling of an enormous aluminum ship floating through the eucalyptus grove on a bed of San Francisco fog, with its prow pointing toward the Pacific Ocean (Image 2). Finally, it seems, the architecture and the science it supports are partners worthy of each other. (Readers may be interested in a recent article on the topic by architecture critic Paul Goldberger in *The New Yorker*, September 19, 2011, p. 88.)

When Viñoly came to town for the building's official opening, I was finally able to slip into his busy schedule. His San Francisco office had assured me that Viñoly enjoyed speaking with scientists, and in fact, I found myself initially being interviewed by *him*. Garbed in black from neck to toe and balancing on his head three pairs of identically framed glasses—one for sun, one for reading, and one for distance—Viñoly was easy to spot. I located him on the bridge that connects my ninth floor office with the new building, just as he was finishing up a conversation with UCSF's Michael Toporkoff, the building's project manager. Let's start by eavesdropping.

**Viñoly:** Are people [in the new building] happy or not?

**Toporkoff:** They are very happy.

**Viñoly:** Take a high rise and put it on its side, and have open areas between the laboratories to promote collaboration.

**Toporkoff:** Tell me, what did you see when you first came to the site? I'm really curious.

**Viñoly:** When we first came to the site, we were appalled that *this* building was [to be] in *this* place!

**Toporkoff:** But the difficulty of the site—did that inspire you to do what you did?

**Viñoly:** Well, this is a building that you couldn't have in any other site in the world. The building *is* the site. But it really does work with a great deal of impact. And functionality is the most important thing—it provides a special place where people can get together and do things that otherwise they couldn't.

I think the gardens are great. At the conference room at the top, it gives you a completely different impression.

**Gitschier:** [Chiming in] So, shall we go to my office?

**Viñoly:** You're on the same floor as the building!

**Gitschier:** I watched this whole building go up, and I must say this has been something! Just prepping the ground—you have these yellow Caterpillar trucks and you think—one wrong move and they are history!

Thank you so much for meeting me.

**Viñoly:** Absolutely my pleasure. Tell me what you are working on.

**Gitschier:** I am a human geneticist, and I won't be going into the stem cell building. I work on a very unusual project on the genetics of absolute pitch, which is the ability to name the pitch of a tone without a reference tone.

**Viñoly:** I know what it is—absolute pitch. I know this very well because I am a musician.

**Gitschier:** What do you play?

**Viñoly:** I play the piano. I play the cello and the flute.

**Gitschier:** How do you have time for all that?

**Viñoly:** I don't, actually. I always have pianos in the offices I go to. I have two pianos in my office in New York, one piano in London. It is the most wonderful thing to do. So I know exactly what absolute pitch is.

Ah, you have Richard Dawkins there [on my bookshelf].

**Gitschier:** I'm starting to do more writing, which is an outgrowth of these interviews, which means I tend to read a lot.

OK, let me ask *you* a few things.

**Viñoly:** But before you do that, what is the logic of absolute pitch? I think it's a very interesting thing. I know very few people with it. One is, amazingly, my sister, who was a piano student and always wanted to sing. And another person who has it is Daniel Barenboim, who is a good friend of mine.

**Gitschier:** How do you know him?

**Viñoly:** Because he is from Argentina and I lived there many years. He has, of course, super training. But I do think, in a funny way, that there is a way of training for that [absolute pitch], too. I can remember sounds that I attribute to one particular tonality. And remembering a sound is a very special thing, really hearing a tone.

**Gitschier:** Well, it's a great topic. Unfortunately, it is totally unfundable.

**Viñoly:** Totally. You are never going to get a penny for it.

**Gitschier:** Right, that's why I need to develop a parallel career!

So, I want to talk about the process you go through and how much you personally, at this point in your life, are involved in the design process. You've done ten or 12 buildings that are research-specific buildings.

**Viñoly:** Probably more than that.

**Gitschier:** What was your first one?

**Viñoly:** The first one was the Van Andel Institute in Grand Rapids, Michigan. It was a wonderful experience, [as it involved] contact with a completely different client. Talking to people who are in science has always been incredibly enlightening for me. I had an aunt who was in neurosciences and who left a mark on me because she was super smart and talked with clarity.

**Gitschier:** Where did she work?

**Viñoly:** She worked also in Uruguay. She was a professor of chemistry and physics at that time in the 1940s. Very famous. And then worked for years in life sciences. I kept that memory of how she looked at things from a completely different perspective.

And we did this first building with Dr. Thomatis, who was in cardiovascular research and was trying to convince the Van Andels, a very wealthy family, that they really needed to invest in science. So he created this idea of the Institute, and David Van Andel picked it up and funded it.

You know, you come to places like yours here, and you get to meet people and people start talking. I always felt that it was an area of intellectual interest that had horrific PR—that you people never quite made it out there to demonstrate how important this work is! But over the years, since we've been working, this has exploded.

**Gitschier:** Do you mean the architectural practice?

**Viñoly:** No, the science practice—biology in general and genetics and all these new fields that are breaking the mold on how people think about disciplines and interactivity between different fields.

In my time, the closest [profession] was to be a pharmacist, and all of sudden, you are in control of practically every single area of development, which has enormous implications in their application and in the knowledge of life. Unbelievable.

**Gitschier:** So, were you commissioned by the Van Andels?

**Viñoly:** It was a competition. At that time—I can't remember exactly when it was—it was a very specialized kind of work. Architects that were of some notoriety never approached it. The fact that you have now some important architects working in this field is the result of the incredible importance that the building type took over time. Because now there is money to support it. But when we started, it was an area of specialists. And I have a difficult time with specialists, in general, because I think architecture isn't a field in which you should focus on one single building type—if you do this, you may lose an ability to look afresh and things may become pigeon-holed or gelled by previous assumptions.

And I noticed from the beginning that people who were working in the field of spaces for scientists were totally off the mark. They didn't listen to what people were saying.

**Gitschier:** That's actually something I want to ask you about, but before we get there, you've done Janelia Farm—I haven't been there.

**Viñoly:** You have not? It is a palace!

**Gitschier:** Ah! Did you have a budget for that?

**Viñoly:** We did have a budget. Actually, it [HHMI] is a very rich institution, but it was a budgeted job, and they did want to promote a model of research that is not university-based and not private-sector-based. And from what I know, it is really functioning quite brilliantly. Dr. Rubin and Dr. Cech had this notion that the Institute needed not just to continue to support the fellows and the researchers, but also create a new way of analyzing problems, and that is what in reality the building is—a new model for how to work together.

**Gitschier:** And how did you get that commission?

**Viñoly:** That was a competition, too. A very strict competition, run by the Institute's architect [Robert McGhee], who is a fabulous man. I worked with him for four years. But again, he had a very clear idea about how to do it, and that was his field of work forever. He worked first for Yale and then, as a result of being the architect of the Institute, every time one of their fellows wanted a new facility somewhere, HHMI provides the funds and the design.

But he had a very special idea and it was an interesting competition because he started by selecting a group of five or six architects, and then he participated with them in a close relationship during the design process for the building. So he was kind of like an internal critic.

We had it completely wrong. He came to the office and told us so. Basically, it was an attempt to develop a series of ideas that I had started to develop at Princeton and other places, and he told us—this was really very close to the deadline of the project—that it was all wrong.

It *was* the wrong scheme, I think. It was too abstract and it had a number of assumptions that in the level of practicality in which he worked, weren't really…

I mean, one of these things that often happens with architects is that you take a tack and can continue going in one direction—I guess in science it is the same thing—and you don't ask whether this is the right way of doing it.

So he was incredibly helpful and completely lethal! Because he told us it wouldn't work.

And then I went back and re-thought. He had a very clear layout that he wanted to almost impose on all the architects—the relationship between the offices and the wet lab and the services and so on.

So I sat down and within a week-and-a-half or so, turned the whole thing around, and did something that he never ever expected. And we didn't have any review because it was so late. And we presented it. I was incredibly convinced that the scheme was magnificent. He was totally surprised!

**Gitschier:** The shock factor worked in your favor.

**Viñoly:** It was a shock, but it was predicated on assumptions that were able to be answered in design terms. It wasn't just the theory of it. In our trade, everything depends on how you do it. I mean this [the UCSF building] is a linear building—but it could be a complete bomb, too. Is it a linear building that steps up and relates to the site and so on?

The same thing happened with the Janelia Farm project. From the beginning, it was such a strong concept, that you create a simple extrusion that steps up. It has the idea of gardens, like your building here. It adjusts very well to the site, the same way that this one does, which is something that I think is very, very important in architecture. The notion that you can come with a pre-conceived idea and plunk it in a site—Jakarta or Bilbao—doesn't seem very sophisticated.

That's the kind of thing that is most important in this building, as in Janelia Farm, because the worst thing that happens in these kinds of buildings is that either you totally disregard what is considered in National Institutes of Health (NIH) terms “scientific space”, and the non-scientific space is the one that gets cut off. In fact, you do as much science in this corridor as in the lab itself, and that's something that can be very easily socially engineered.

To me, the building is a platform for the people who use it. It has the ability to determine the way you walk, the way you relate to things, adjacency—all of those things are important. But the most important thing, in my mind, is the fact that there is a connection between the quality of the space and the quality of the thinking. Which is something that people have always put on the back burner.

You shouldn't be working in a building that has a “wow” moment. You know the building in San Diego? Not Salk.

**Gitschier:** Scripps?

**Viñoly:** Have you entered? You see this enormous atrium at the entrance—that is supposed to be for effect. But then all the other spaces are very much normal. I think that creating a spatial structure that gives people the ability to locate themselves within the building—a place where they have a journey to make, to meet other people, to say “Hello” in the morning—is important. Think of the scientific activity—that is just as dependent on the quality of the space as it is on the quality of the equipment.

You've been at NIH. The old labs are like cells. Like torture chambers! And I think that [the change in design since then] is important. Because the science has developed so rapidly and strongly, people are really more vocal. They ask for things that people weren't conscious of before.

I think the next phase is a much more intriguing phase to be in the design of these buildings—the rigidity of the building envelope is still something that needs to be shaken up. In other words—look at your office, it's cluttered with things.

**Gitschier:** Hey, my office is pretty neat compared to some!

**Viñoly:** But if you could somehow rearrange these things yourself…which I'm sure you do every so often when the stuff covers you and you can't walk in.

That type of situation in a lab space is something that shouldn't be that difficult to achieve. The flexibility of the hardware should be something that the building itself is capable of doing, as opposed to just walking in and having to live with that forever. It sounds like something that belongs to another technological era. In the construction trade, we still build like the [ancient] Egyptians. The technology should be able to provide us with a much more open-ended environment. You don't need to ask anyone to move this table. There isn't much difference between this table and a wet lab. You should be able to configure your working space in practical manner. That to me is what is next. How to make the building much more like a tool.

**Gitschier:** Let's take an example. You were invited to do the design of our new building. What is the first thing you do? Do you come out to see the space? Do you meet the people who will be residents of the space?

**Viñoly:** The first thing you do is look at the preconception the client has about the building. In any architectural project there is a condition that, as a client, you always sort of know what you want. And I always think that what a client always thinks he or she wants is *wrong*.

**Gitschier:** Is wrong. OK. So in this case, did UCSF say something that you assumed would be wrong?

**Viñoly:** Yes.

**Gitschier:** What was it?

**Viñoly:** They got very nervous! I said that the site was a very difficult site, not only because it is difficult topographically and location-wise, but also because it was in the back of the house for a science [stem cell research] that was supposed to be on the forefront of everything else. One of the things we did was transform this thing into the “front” by creating an interesting connection with the topography, by opening it to views, by reinforcing the presence of the street, which is one way you can access it.

**Gitschier:** So did you physically come out here at that initial point?

**Viñoly:** Yes. So the first part of this exercise was that. And this was an interview process because this was also kind of like a competition.

**Gitschier:** It wasn't a sealed deal yet.

**Viñoly:** No. And we showed a series of ideas that were unique and very different. We said to them that we thought the building was too important for the site, that it needed something! And I showed something, which was pretty much this, and it was pretty much outside the site.

**Gitschier:** Oh, you made it bigger than the site that they had planned on! When I saw the extent that this was going to cover, I couldn't believe it—it is going on forever. Although I did have some small appreciation of it from the renderings, I just didn't realize the extent.

You're the principal of this huge architectural firm.

**Viñoly:** Not so huge.

**Gitschier:** Were these your own plans? Do you do this with a pen and paper?

**Viñoly:** Yes. We build a physical model. Of the mountain—the site. We look at it, and we say we have to do something other than we were asked to do.

**Gitschier:** And they bought it?

**Viñoly:** The scientists bought it. But it was an interesting interview because it was very much off the wall. I said, “You have to forgive me, but I think this has got to be this big.”

So their eyes went like this! So the university is on board and the scientists are on board. Then all the trouble starts.

**Gitschier:** It does?

**Viñoly:** Because it is a very difficult site. There is no site!

**Gitschier:** Let me ask you about the other end. Once a building is finished, everybody moves in. Two questions. Do you get feedback from the residents? Do you go back and visit so that you can learn from it?

**Viñoly:** This is one thing we *do* do. The most important thing in this type of work is not so much the critical acclaim, but whether the building becomes the property of the people who use it. And I guess we're very, very lucky with that because the important buildings for me—that I have done—are important because they have increased the coefficient of happiness. And I think that is something that you don't find very often in important pieces of architecture. People have to fight the building.

**Gitschier:** And you don't want people to do that.

**Viñoly:** I don't think there is enough time in life for that.

**Gitschier:** Then part b of my final question: Are you horrified? We people who work in a lab generally have no design aesthetics.

**Viñoly:** You would be surprised! But I know what you mean.

**Gitschier:** I would say generally labs end up looking pretty disgusting—there are post-its, cords, chemical spills. Are you horrified then when you go back?

**Viñoly:** Absolutely not! I think it is the greatest thing!

**Gitschier:** Oh! I would imagine you would think, “I have this beautiful line…”

**Viñoly:** Quite the opposite. I'm too old for that. You see, this is a completely different way of looking at the importance. I mean, if your aesthetic contribution to architecture hinges on the fact that people cannot move a piece of furniture because it's aligned with something else, then that's a pretty sad prospect. Quite the opposite. All of these buildings encourage that type of appropriation. The problem is that the mess, as you call it, is always generated by the limitations that the building imposes on you, when instead the building should encourage you. The ability to link things in a walkable condition, for example. All of those things are registered by people. I don't believe that you need to be an architectural critic for that. In fact, if you are an architectural critic you are definitely wrong about this.

People do remember and relate to the absolutely basic architectural principles, which are that you are in a site that is absolutely spectacular; that what was supposed to be the back of the house is now the front of the house—a thoroughly unexpected condition; that is without comparison with any other buildings around here, or on the rest of campus for that matter. And those things are incredibly important and are the core of the success of the building, in my mind.

As I said, a roof could be a roof, but it also could be a little garden. You haven't spent too much money in doing it, and adding to the functions that are typical in an environment like this. But it is a very subtle thing. It doesn't depend on the awkwardness of the shape. You don't need to force things. This looks like an interesting building, but it wasn't thought out as a form. It was thought out as an organism.

The distinction between the building as sculpture or the building as a functional element, for me, is a completely artificial dilemma. Architecture is about how the working of the building is capable of improving your own attitude towards the work. And for a building where you do science this is paramount.

**Gitschier:** So when you come out here and look at this building, are you pleased with the result?

**Viñoly:** I think I am. But it's impossible to be completely happy about everything. Our building had very important limitations in budget. There are many things that I would have done differently.

And that is an important thing too. The success of a building shouldn't depend on the way you solved a very peculiar part of the architecture. There was a piece of material that was to be part of the underside of the building. The success of the building doesn't depend on it.

**Gitschier:** Well, I'm just thrilled that you were able to spend some time.

**Viñoly:** Listen, sorry for this. I would rather stay here. [Checking out a messy array of photos on his way out] Is this your family?

**Gitschier:** This is my dad, who just died. This is my sister and my daughter.

**Viñoly:** Oh, how lovely.

